# Novel application of Roux-en-Y for diversion of a cutaneous fistula from an irresectable mesenteric root mature teratoma: a case report

**DOI:** 10.1093/jscr/rjad630

**Published:** 2023-11-21

**Authors:** Xavier Field, Fraser Welsh

**Affiliations:** Department of General Surgery, Te Whatu Ora Health New Zealand Waikato, 183 Pembroke Street, Hamilton 3240, New Zealand; Department of General Surgery, Te Whatu Ora Health New Zealand Waikato, 183 Pembroke Street, Hamilton 3240, New Zealand

**Keywords:** mature teratoma, Roux en Y, mesenteric root, cystojejunostomy, cutaneous fistula

## Abstract

Reconfiguration of the alimentary tract with the Roux-en-Y has been utilized in a wide variety of contexts since its first description by Swiss physician César Roux. We present a novel and unique application of the Roux-en-Y whereby a chronically discharging cutaneous fistula originating at a retroperitoneal mature teratoma within the root of the mesentery was diverted enterically via a cystojejunostomy and the fistula tract excised, providing a resolution of symptoms. The location of the tumour in the root of the mesentery and the involvement of major mesenteric vessels made a radical resection of the tumour technically impossible but due to the distressing symptoms caused by the fistula made diversion of the fistula an excellent treatment option.

## Introduction

Reconfiguration of the alimentary tract with the Roux-en-Y has been utilized in a wide variety of contexts since its first description by Swiss physician César Roux [1]. Diversion and bypass of gastrointestinal contents occurs whilst overall continuity of the gastrointestinal tract is maintained. Herein, we present a unique application of the Roux-en-Y where a chronically discharging cutaneous fistula originating at an irresectable retroperitoneal mature teratoma within the root of the mesentery was diverted enterically via a cystojejunostomy and the fistula tract excised, providing a resolution of symptoms.

## Case report

In December 2020, a 29-year-old man presented to the surgical outpatient clinic with a discharging sinus from a previous subcostal rooftop incision. Previously in October 2020, he had had an emergency laparotomy and attempted resection of a large mesenteric lesion that was splaying his mesenteric vessels. This had been found incidentally on imaging of his spine done due to discitis. The radiologic appearance of the lesion was in keeping with a teratoma.

**Figure 1:**
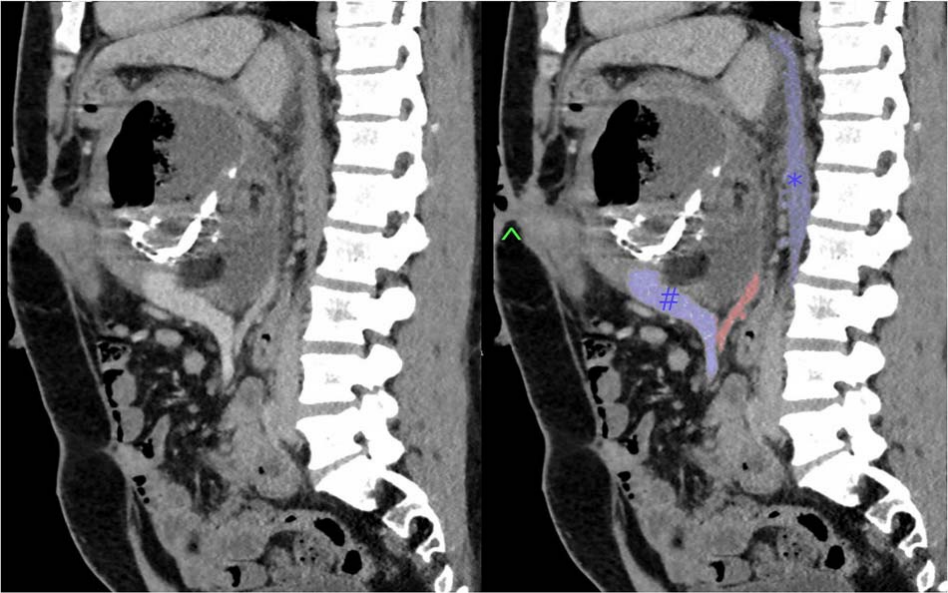
Sagittal section of pre-operative CT with portal venous contrast showing the lesions relationship to major vascular structures of the abdomen. Labels: ^ - fistula opening, blue # - superior mesenteric vein, blue * - inferior vena cava, red - superior mesenteric artery.

Subsequently, the patient developed severe abdominal pain and a computed tomography (CT) demonstrated possible impending perforation of the teratoma. Emergency laparotomy was performed. The teratoma was found posterior to the neck of the pancreas extending retroperitoneally behind the lesser sac. The portal structures and pancreas were attenuated and stretched over the lesion. Both the superior mesenteric vein (SMV) and inferior mesenteric vein were densely adherent to the teratoma. The superior mesenteric artery (SMA) was not able to be visualized (Fig. 1). An initial attempt was made at resection of lesion. However, when beginning to mobilize the lesion it was felt that, in order for a complete resection, at least a total pancreatectomy with portal vein reconstruction was to be required. However, due to the fact that it may not be possible to resect the lesion from the SMA or the possibility that the portal vein and SMV could not be anastomosed, radical resection was abandoned. Instead, a debulking of the mass was performed with drainage of perforated cystic contents from the abdomen.

Histology revealed a cystic structure partly lined by skin with adnexae and partly by columnar and cuboidal epithelium. Mature apidose tissue was seen in the cyst wall as well as patchy areas of salivary acinar tissue. No immature tissues were seen.

Two months later the patient presented to the outpatient department with yellow gelatinous discharge from his wound. The patient’s quality of life was being significantly hampered by this. The discharge was partially controlled by placing a stomal apparatus over the sinus but this would often dislodge and the patient resorted to sleeping with a towel over his abdomen to collect the discharge.

**Figure 2:**
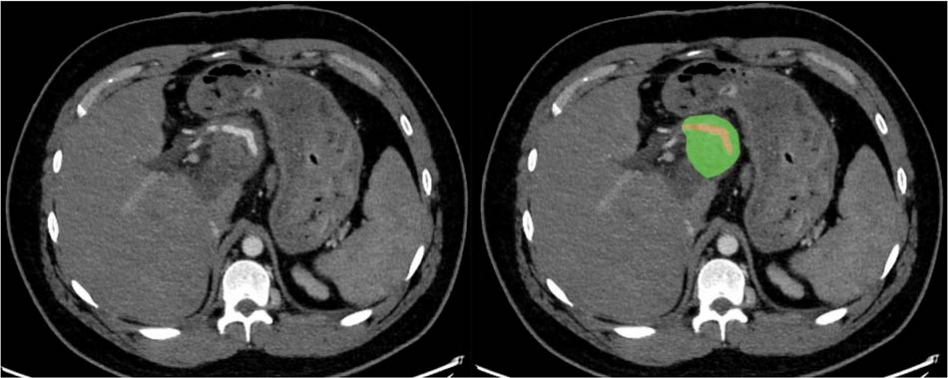
Axial section of CT with arterial contrast showing the hepatic artery (red) traversing within the superior aspect of the lesion (green).

**Figure 3:**
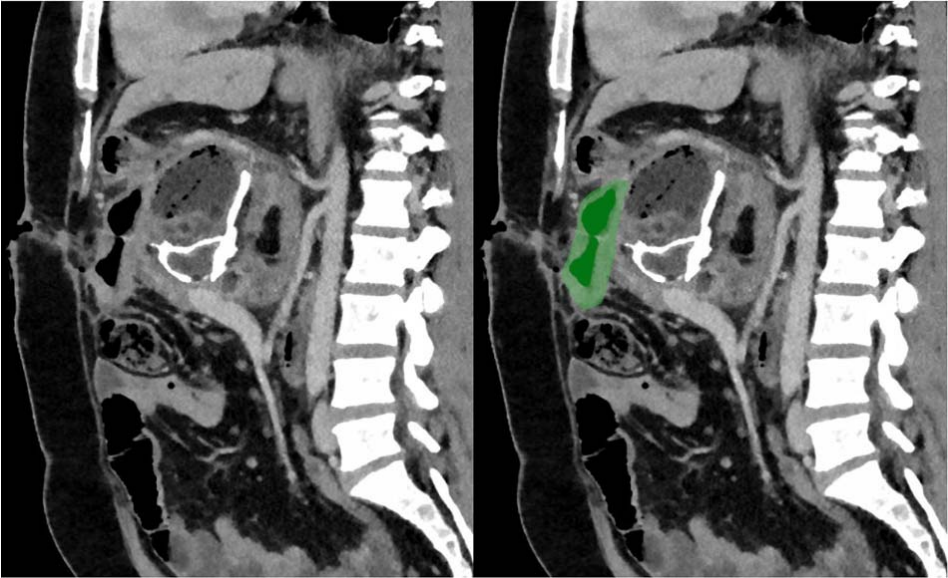
Sagittal section of post-operative CT with portal venous contrast demonstrating cystojejunustomy, highlighted in green, anastomosed to the origin of the fistula.

Due to the debilitating nature of the patient’s condition and his wishes for an attempt at treatment, he was brought forward for a further exploratory laparotomy. The patient’s rooftop incision was reopened and the wound and sinus tract were excised *en bloc*. The stomach and transverse colon were found to be densely adherent to the teratoma. These were carefully mobilized and dissected free from the teratoma. Again, resectability of the teratoma was assessed and deemed to be impossible due to the hepatic artery being inseparable from the lesion (Fig. 2). Instead, internal drainage with diversion of the sinus discharge via a jejunostomy with a Roux en Y reconstruction of the alimentary tract was performed. The jejunum was divided at 50 cm from the duodenojejunal (DJ) flexure and the Roux limb was brought up to the origin of the sinus through a window made in the mesentery of the transverse colon. The lumen was opened and sutured over the origin of the sinus with 4/0 PDS sutures (Fig. 3). Finally, a jejunojejunostomy was formed to return continuity to the jejunum.

The patient recovered well and was discharged on the fifth post-operative day. He subsequently represented to hospital with a superficial wound infection, which was treated with laying opening of the superficial tissues and application of negative pressure wound therapy. He has been subsequently follow up at 7 months post-operative and there has been no further offensive discharge.

## Discussion

Teratomas are germ cell tumours constituting at least two of the three germ cell layers: endoderm, mesoderm, or ectoderm [2]. They can occur either within the gonads or extragonadally. Extragonadal teratomas most often occur in the midline of the body, in the sacrococcygeal region, mediastinum, retroperitoneum, among others [3–7].

Mature teratomas contain adult-type, well-differentiated cells. The natural history of mature teratomas is commonly benign, however, these tumours can have malignant and metastatic potential [2, 8]. Definitive diagnosis is only achieved with complete histological evaluation. Thus, surgical resection is required for diagnosis and treatment. The size and volume of teratoma will increase over time and a local pressure effect can be exerted on surrounding viscera, as such they can grow to enormous size in locations such as the retroperitoneum.

A parallel application of internal drainage with a Roux-en-Y reconfiguration can be seen in the management of pancreatic pseudocysts. Although, more recently commonly drained endoscopically, surgical cystogastrostomy or cystojejunostomy is well described in the literature as treatment of pseudocysts [9].

This case presented a significant management challenge to the treating team. The distressing nature of the symptoms from the discharging sinus tract meant finding an operative solution was imperative. Due to the nature of the teratoma, fistula output would not cease without resection of the cystic components. However, the location of the tumour in the root of the mesentery and the involvement of major mesenteric vessels made a radical resection of the tumour technically impossible. Cystogastrostomy and cystoduodenostomy were considered but due to the size of the tumour, neither the stomach nor duodenum would be able to be anastomosed to the origin of the sinus in a tension-free manner. Another benefit of the Roux en Y configuration was the ability to keep the origin of the sinus separate from the alimentary stream limiting the chance of reflux of the tumour contents. This novel utilization of Roux en Y internal diversion provided an excellent option for treatment of the sinus.
